# *LIN28B*, *LIN28A*, *KISS1*, and *KISS1R *in idiopathic central precocious puberty

**DOI:** 10.1186/1756-0500-4-363

**Published:** 2011-09-22

**Authors:** Johanna Tommiska, Kaspar Sørensen, Lise Aksglaede, Rosanna Koivu, Lea Puhakka, Anders Juul, Taneli Raivio

**Affiliations:** 1 Institute of Biomedicine/Physiology, University of Helsinki, Helsinki, Finland; 2 Children's Hospital, Helsinki University Central Hospital (HUCH), Helsinki, Finland; 3 Department of Growth and Reproduction, Copenhagen University Hospital, DK-2100 Copenhagen, Denmark

**Keywords:** *LIN28B*, *LIN28A*, *KISS1*, *KISS1R*, idiopathic central precocious puberty, timing of puberty

## Abstract

**Background:**

Pubertal timing is a strongly heritable trait, but no single puberty gene has been identified. Thus, the genetic background of idiopathic central precocious puberty (ICPP) is poorly understood. Overall, the genetic modulation of pubertal onset most likely arises from the additive effect of multiple genes, but also monogenic causes of ICPP probably exist, as cases of familial ICPP have been reported. Mutations in *KISS1 *and *KISSR*, coding for kisspeptin and its receptor, involved in GnRH secretion and puberty onset, have been suggested causative for monogenic ICPP. Variation in *LIN28B *was associated with timing of puberty in genome-wide association (GWA) studies. *LIN28B *is a human ortholog of the gene that controls, through microRNAs, developmental timing in *C. elegans*. In addition, *Lin28a *transgenic mice manifest the puberty phenotypes identified in the human GWAS. Thus, both *LIN28B *and *LIN28A *may have a role in pubertal development and are good candidate genes for monogenic ICPP.

**Methods:**

Thirty girls with ICPP were included in the study. ICPP was defined by pubertal onset before 8 yrs of age, and a pubertal LH response to GnRH testing. The coding regions of *LIN28B, LIN28A, KISS1*, and *KISS1R *were sequenced. The missense change in *LIN28B *was also screened in 132 control subjects.

**Results:**

No rare variants were detected in *KISS1 *or *KISS1R *in the 30 subjects with ICPP. In *LIN28B*, one missense change, His199Arg, was found in one subject with ICPP. However, this variant was also detected in one of the 132 controls. No variation in *LIN28A *was found.

**Conclusions:**

We did not find any evidence that mutations in *LIN28B *or *LIN28A *would underlie ICPP. In addition, we confirmed that mutations in *KISS1 *and *KISS1R *are not a common cause for ICPP.

## Background

During the last decades, a decline in age at pubertal onset in girls has been reported worldwide [1,2], paralleled by an increase in the incidence of idiopathic central precocious puberty (ICPP) [3]. However, the etiology underlying the precocious reactivation of the hypothalamic-pituitary-gonadal (HPG) axis in girls with ICPP is still largely unresolved. As compared with normal-timed pubertal controls, girls with ICPP have increased adiposity and decreased insulin sensitivity at diagnosis, suggesting a causal link between these metabolic factors and HPG axis programming [4]. However, these metabolic factors may only elicit an early HPG activation in the presence of a susceptible genetic background. Overall, the genetic modulation of pubertal onset most likely arises from the additive effect of multiple genes [5], but also monogenic causes of ICPP probably exist, as cases of familial ICPP have been reported (reviewed in [5]). For example, a 27.5% prevalence of familial ICPP has been reported in one study; the suggested mode of inheritance was autosomal dominant with incomplete sex-dependent penetrance [6].

Only few genes have been investigated in mutation screening studies in ICPP. Inactivating mutations in the *KISS1R *(*GPR54*) gene cause autosomal recessive normosmic idiopathic hypogonadotropic hypogonadism (nIHH) [7]. *KISS1R *codes for a G protein-coupled receptor which with its ligand, kisspeptin, forms an excitatory neuroregulator system for GnRH secretion. Interestingly, an autosomal dominant activating *KISS1R *mutation has been described in a girl with ICPP [8], and very recently, also mutations in *KISS1*, coding for kisspeptin, were suggested to underlie ICPP [9]. Thus, inactivating or activating mutations in the same key genes governing the HPG axis function may have respective effects on the phenotype. The role for *KISS1R *and *KISS1 *in ICPP has also been suggested in association analyses [10,11]. However, so far none of these results has been verified or replicated in other study populations. Especially, the frequencies of *KISS1R *and *KISS1 *mutations in other series of ICPP patients have not been reported.

Variation in or near the *LIN28B *gene has been found associated with age at menarche in four independent genome-wide association (GWA) studies [12-15], and, in one of them, the allele associated with earlier age at menarche was also associated with other markers of pubertal timing (earlier breast development; earlier voice breaking and more advanced pubic hair development; faster tempo of height growth and shorter adult height) [13]. We subsequently investigated whether mutations in the coding region of *LIN28B *could account for delayed puberty in a large, well-phenotyped cohort of subjects with constitutional delay of growth and puberty (CDGP), but no mutations were found [16]. *LIN28B *is a human ortholog of the gene that regulates processing of microRNAs (miRNAs) which control the timing of major developmental events in *C. elegans*: gain-of-function and loss-of-function mutations result in retarded or precocious development, respectively [17]. In addition, *Lin28a *(functionally redundant homolog of *Lin28b*) transgenic mice manifest the puberty phenotypes identified in the human GWA studies [18]. Thus, both *LIN28B *and *LIN28A *may have a role in pubertal development and are good candidate genes for monogenic ICPP.

Here, we investigated mutations in *LIN28B*, *LIN28A*, *KISS1*, and *KISS1R *in 30 girls with ICPP.

## Methods

### Subjects

Thirty girls with ICPP were recruited from our outpatient clinic at the Department of Growth and Reproduction, Copenhagen University Hospital, Denmark. Details of this study have previously been published [4]. All patients presented with breast budding as first sign of puberty, and all were pre-menarcheal at diagnosis. The girls were diagnosed with ICCP if the following criteria were met: age at onset of breast development < 8 yrs, peak-LH level > 5 IU/l in response to rapid-acting GnRHa (0.1 mg of Relefact^® ^LH-RH), and a non-pathological brain MRI. In addition, bone age and sex steroid hormones were evaluated. The mean age at onset of puberty was 7.5 yrs (6.5-7.9) with a mean bone age advancement of 1.4 yrs (-0.1-2.8). One hundred thirty-two healthy controls were recruited from The COPENHAGEN Puberty Study. This study has previously been described in details [1,19]. In brief, healthy girls and boys were recruited from primary schools in the Copenhagen area from May 2006 to January 2008. In total, 995 healthy Caucasian girls participated. Of these, the present sample represents all prepubertal girls aged 8.1-9.9 yrs with available DNA. Pubertal development (breast stage B1-B5 and pubic hair stage PH1-PH5) was described according to Tanner's criteria supplemented with breast palpation to better discern true glandular breast tissue from adipose tissue.

### Mutation analysis

Genomic DNA from peripheral blood leukocytes of the subjects was extracted. The coding exons and the exon-intron boundaries of *LIN28B*, *LIN28A*, *KISS1*, and *KISS1R *were PCR amplified from all subjects with ICPP. In addition, exon 4 of *LIN28B *was screened in DNA samples from 132 healthy control subjects. PCR products were purified with ExoSAP-IT treatment (Amersham Biosciences, Piscataway, NJ, USA), and sequenced using the ABI BigDyeTerminator Cycle Sequencing Kit (v3.0) and ABI 3730xl 96-capillary DNA Analyzer automated sequencer (Applied Biosystems, Foster City, CA, USA). Sequences were aligned and read with Sequencher^® ^4.9 software (Gene Codes Corporation, Ann Arbor, MI, USA). All primer sequences and PCR conditions are available upon request.

### Ethics

The study was approved by the local ethical committee (# KF 01 282214 and KF 11 2006-2033), and the Danish Data Protection Agency. All participants and their parents gave informed consents.

## Results and Discussion

Only one sequence variant was found in the coding region of *LIN28B*; a missense change c.596A > G (p.His199Arg) (rs118000887) was detected in a girl with ICPP: her age at pubertal onset was 7.8 yrs, she was in Tanner stage B3 and PH2 at diagnosis (8.9 yrs), had bone age advancement of 2.1 yrs, and gonadotropin and estradiol levels consistent with an early activation of the HPG axis (plasma estradiol 131 pmol/L; FSH 5.11 IU/L; LH 2.04 IU/L). This His199Arg change was predicted to be possibly damaging by PolyPhen http://genetics.bwh.harvard.edu/pph/[20,21], but it was also present in 1 of the 132 controls: this girl had Tanner stage B1 and PH1 at the age of 8.9 yrs, and she did not deviate from the other controls with respect to height, weight and BMI, as well as gonadotropins, sex steroids, SHBG, or IGF-1 levels (all her values were within the mean ± 2 SDs of the controls). Thus, this missense change is not likely to be causative for ICPP, and it is not of clinical value in the early detection of girls at risk of ICPP. No sequence variation was detected in the coding region of *LIN28A*, a finding in accordance with the rarity of reported polymorphisms in this area http://www.ensembl.org/index.html[22]. We do realize that our sample size is small. However, if mutations in the coding region of *LIN28B *or *LIN28A *did cause 15% of ICPP, we would have with 99% probability found at least one mutation in 30 subjects. We can thus conclude that mutations in *LIN28B *and *LIN28A *are not at least a common cause for ICPP. Of course, as only coding regions of these genes were sequenced, there may be mutations outside these regions, not detected in our study. Especially, there are miRNA regulatory elements in the 3'UTR of *LIN28B*, mutations in which could affect the function of the gene and have an impact on the timing of puberty; in CDGP, however, this was not the case [16]. Involvement of *LIN28B *and *LIN28A *in the regulation of pubertal timing in humans still warrants further studies in different patient groups and populations.

In *KISS1 *and *KISS1R*, our cases carried only common previously known single nucleotide polymorphisms (SNPs) http://www.ensembl.org/index.html[22]. Given the central role of the kisspeptin-KISS1R signaling complex in the pubertal activation of GnRH neurons and the reproductive axis [7], a defective kisspeptin system appears to be an obvious candidate in the pathogenesis of sexual precocity. An autosomal dominant missense mutation, Arg386Pro, in *KISS1R *(*GPR54*), leading to prolonged activation of intracellular pathways in response to kisspeptin, has been suggested to cause central precocious puberty in an adopted girl whose biologic family was not available for genetic studies [8]. To the best of our knowledge, no other ICPP cases with activating *KISS1R *mutations have been reported. Recently, Silveira *et al. *[9] studied 83 children (77 girls) with ICPP for mutations in *KISS1*, and reported two missense variants, c.369C > T (p.Pro74Ser) and c.417C > G (p.His90Asp), in three unrelated children: two unrelated girls with sporadic ICPP were homozygous carriers of the variant His90Asp, and one boy with sporadic ICPP had a heterozygous Pro74Ser. These variants were not detected in 200 controls, but only Pro74Ser differed from the wild type in the *in vitro *studies: the variant appeared to result in higher kisspeptin resistance to degradation. However, the proband's mother and maternal grandmother were also carriers of the variant, but had menarche at appropriate ages. No mutations in *KISS1 *were found in 101 Korean girls with ICPP [23]; only known polymorphisms or synonymous changes were detected. In accordance, no mutations were found in either *KISS1R *or *KISS1 *in our 30 girls with ICPP, suggesting that defects in the kisspeptin system are a rare cause for ICPP. We cannot, however, rule out the possibility that mutations outside the coding regions, for example in the regulatory regions of these genes, could be found in these patients.

## Conclusions

Our results suggest that mutations in the coding region of *LIN28B *or *LIN28A *do not play a major role, if any, in the genetic etiology of ICPP. In addition, we confirmed that mutations in *KISS1 *and *KISS1R *are not common causes of ICPP in girls.

## List of abbreviations

ICPP: idiopathic central precocious puberty; GWA study: genome-wide association study; HPG axis: hypothalamic-pituitary-gonadal axis; nIHH: normosmic idiopathic hypogonadotropic hypogonadismi; CDGP: constitutional delay of growth and puberty; miRNA: microRNA; SNP: single nucleotide polymorphism

## Competing interests

The authors declare that they have no competing interests.

## Authors' contributions

JT participated in the study design, planned the molecular genetic studies, and drafted the manuscript. KS and LA collected the patient samples and clinical data in the study, and helped to draft the manuscript. RK and LP carried out most of the molecular genetic studies. AJ and TR participated in the study design and helped to draft the manuscript. All authors read and approved the final manuscript.

## Funding

This work was supported by the Kirsten and Freddy Johansen Foundation, the Danish Medical Research Council, the European Union (DEER, grant 212844), the Academy of Finland, the Foundation for Paediatric Research, the Helsinki University Central Hospital Research Funds, the Sigrid Juselius Foundation, Emil Aaltonen Foundation, Novo Nordisk Foundation, Jalmari and Rauha Ahokas Foundation, Finnish Cultural Foundation, and Helsinki University Research Funds.
